# Abdominal Wall Mycetoma Presented as Obstructed Incisional Hernia of Cesarean Section in Eastern Sudan

**DOI:** 10.1155/2007/74643

**Published:** 2007-03-07

**Authors:** Osama A. Elhardello, Elsadig S. Adam, Ishag Adam

**Affiliations:** ^1^New Halfa Teaching Hospital, P.O. Box 61, New Halfa, Sudan; ^2^Department of Obstetrics and Gynaecology, Faculty of Medicine, University of Khartoum, P.O. Box 102, Khartoum, Sudan

## Abstract

Mycetoma a worldwide disease frequently occurs in the tropics with the highest prevalence being in Africa. *Madurella mycetomatis* is the main causative organism of human eumycetoma in Sudan. The legs and feet were commonly the sites of the infection. A 22-year-old lady was presented with painful abdominal swelling around a previous caesarian section scar. A provisional diagnosis of obstructed incisional hernia was put. Histopathological examination revealed macroscopically four masses of soft tissue. Microscopic sections showed grains of *Madurella mycetomatis*.

## 1. INTRODUCTION

Mycetoma is a worldwide disease that frequently occurs in the tropics with the highest prevalence being in Africa [1]. *Madurella mycetomatis* is the main causative organism of human eumycetoma in Sudan [2]. The site of involvement varies with the casual agent and the geographical distribution [3]. The disease is characterized by extensive subcutaneous masses, usually with sinuses draining pus and fungal grains. The characteristic triad, of a subcutaneous mass, sinus, and the presence of discharge containing grains are pathognomonic of mycetoma. It usually involves the subcutaneous tissue after a traumatic inoculation of the causative organism. Feet and legs were usually the sites of the infections, but rare sites were reported too. Hereby, we presented abdominal wall mycetoma presented as obstructed incisional hernia of cesarean section in eastern Sudan.

## 2. CASE REPORT

A 22-year-old lady was presented with an abdominal swelling—around a previous caesarian section scar—gradually increasing in size for one year. In the last 2 days, it became tense, increased in size, and very painful. There was no vomiting or abdominal distention and she had normal bowl habits. She had mild fever but no otherwise significant symptoms. There was no past history of trauma or thorn prick injury to the site of the swelling and no other body swellings. She was not known diabetic or having any other chronic illness.

On examination, she looked ill but not pale nor emaciated. Her pulse rate was 100 per minute, her temperature was 38°C. Her abdomen was not distended; there was a subumblical midline scar. There was a swelling involving the umbilical region and encroaching the hypogastrium about 5 × 8 cm, the swelling was tense, and did not increase in size with coughing, skin over it was normal. It was tense, tender, hot, firm in consistency, and nonreducible. There was no organomegaly and bowl sounds were heard normal.

A provisional diagnosis of obstructed incisional hernia containing an omentocele was put and the patient was prepared for surgery. Her hemoglobin was 10.5 g/dl, her urine was clear, and her renal and liver function tests were within normal range.

There was no peritoneal sac identified intraoperatively; instead a nodulated mass which involved the anterior and posterior walls of the rectus sheath together with the rectus abdominis muscle. The peritoneum was intact. On dissecting the mass, pus was found trickling from cavities containing black grains. The mass was excised with a margin of safety, the field was soaked with iodine tincture 2%, and the rent was closed. The patient was put on ketoconazole tablets 400 mg/d for 6 months to be followed in the referred clinic.

Histopathological examination revealed macroscopically 4 masses of soft tissue measuring 1, 2, 4.5, and 4 cm. Microscopic sections showed grains of *Madurella mycetomatis*, surrounded by heavy neutrophilic inflammatory cells (type 1 reaction).

## 3. DISCUSSION

Mycetoma has been a disease of major concern during the last few decades, in areas where it is endemic. Its importance came from being a significant cause of morbidity and mortality. The disease is supposed to originate from traumatic inoculation of plant material or soil contaminated by these fungi [2]. It has a male predominance, commonly affecting adults between 20–40 years of age and these are the earning members of the society especially in developing countries. The commonest site for mycetoma is the foot, the hand is the second commonest site [4]. However, rare sites such as the chest, abdominal walls, fascial bones, mandible, paranasal sinuses, eyelid, vulva, orbit, scrotum, and surgical incisions may be affected [5, 6]. Primary actinomycosis of the anterior abdominal wall is rare [7].

In this case, the course of transmission of the organism is questionable and difficult to predict. It could be an iatrogenic cause resulting from peroperative introduction of the organism into the tissues. As it could be the result of pre- or postoperative inoculation although the patient has no history of any trauma to the region of the swelling. Yet, it remains to be elucidated in view with other cases reported in likewise parts of the body.
